# A Web-Based Course on Public Health Principles in Disaster and Medical Humanitarian Response: Survey Among Students and Faculty

**DOI:** 10.2196/mededu.8495

**Published:** 2018-01-26

**Authors:** Greta Tam, Emily Ying Yang Chan, Sida Liu

**Affiliations:** ^1^ Jockey Club School of Public Health and Primary Care The Chinese University of Hong Kong Hong Kong China (Hong Kong); ^2^ Collaborating Centre for Oxford University and Chinese University of Hong Kong for Disaster and Medical Humanitarian Response The Jockey Club School of Public Health and Primary Care The Chinese University of Hong Kong Hong Kong China (Hong Kong)

**Keywords:** disaster planning, online education, Donabedian model, public health

## Abstract

**Background:**

Web-based public health courses are becoming increasingly popular. “Public Health Principles in Disaster and Medical Humanitarian Response” is a unique Web-based course in Hong Kong. This course aimed to fill a public health training gap by reaching out to postgraduates who are unable to access face-to-face learning.

**Objective:**

The aim of this paper was to use a structured framework to objectively evaluate the effectiveness of a Web-based course according to Greenhalgh et al’s quality framework and the Donabedian model to make recommendations for program improvement.

**Methods:**

An interim evaluation of the first cohort of students in 2014 was conducted according to the Donabedian model and a quality framework by Greenhalgh et al using objective and self-reported data.

**Results:**

Students who registered for the first cohort (n=1152) from June 16, 2014 to December 15, 2014 (6 months) were surveyed. Two tutors and the course director were interviewed. The Web-based course was effective in using technology to deliver suitable course materials and assessment and to enhance student communication, support, and learning. Of the total number of students registered, 59.00% (680/1152) were nonlocal, originating from 6 continents, and 72.50% (835/1152) possessed a bachelor’s or postgraduate degree. The completion rate was 20.00% (230/1152). The chi-square test comparing students who completed the course with dropouts showed no significant difference in gender (*P*=.40), age (*P*=.98), occupation (*P*=.43), or qualification (*P*=.17). The cost (HK $272 per student) was lower than that of conducting a face-to-face course (HK $4000 per student).

**Conclusions:**

The Web-based course was effective in using technology to deliver a suitable course and reaching an intended audience. It had a higher completion rate than other Web-based courses. However, sustainable sources of funding may be needed to maintain the free Web-based course.

## Introduction

### Web-Based Public Health Courses

With the recent advances in Internet connectivity and increased mobile phone usage, Web-based public health courses have become increasingly convenient and popular. These vary from accredited courses such as Web-based master’s and doctoral degrees to credit-free courses such as the massive open online courses (MOOC), which became popular in 2011 after Stanford University launched its first MOOC [1]. A single MOOC can have enrollment exceeding 100,000 students [2]. The majority of schools accredited by the Council on Education for Public Health offer Web-based courses [3].

Web-based courses offer opportunities for flexible learning, as students are not restricted to learn at fixed times and places. Cost of travel, living expenses, and tuition are reduced as compared with on-site courses. Students may also benefit from exposure to peers from a wider range of global backgrounds. These advantages may be especially appealing to those working in disaster settings, often with irregular schedules in developing countries. The University of South Florida College of Public Health offers numerous Web-based courses but reported that the course in global disaster management and humanitarian relief grew most quickly in popularity [1].

### “Public Health Principles in Disaster and Medical Humanitarian Response” Web-Based Course

This is a free 6-month program offered to anyone with interest in disaster and medical humanitarian response, although it is aimed at postgraduate level. All material is available on the Web and is completed independently at each participant’s desired pace. Support from fellow students is available through online forums, and tutors answer queries via email. Program milestones consist of 4 formative quizzes and 1 final quiz, where a minimum score is required for course progression and certificate of course completion. Table 1 describes the program schedule.

### Effectiveness of the Web-Based Course

A meta-analysis by the US Department of Education reported that purely Web-based learning is as effective as classroom instruction. Most studies surveyed students’ demographics, knowledge, satisfaction, and completion rates [4,5]. Although these criteria are useful for comparing online learning with classroom instruction, they are insufficient for comprehensively evaluating online learning. Web-based courses encounter differing levels of participation and completion. Many students participate in MOOCs, but the completion rate is only 7.0% to 9.0% [6]. This may be due to potential barriers negatively affecting students’ experience of online learning, such as technical problems, decreased instructor and peer presence, and difficulties in time management and self-directed learning [7]. These problems may be further exacerbated by the wide range of student backgrounds in education, culture, technical access, and time. There is lack of framework for standardized evaluation of Web-based courses. The Quality Assurance Agency for Higher Education does not assess MOOCs, as they are noncredit bearing and have no entry requirements [8]. The objective of this study was to use a structured framework to objectively evaluate the effectiveness of a Web-based course according to Greenhalgh et al’s quality framework and the Donabedian model to make recommendations for program improvement.

### Source of Data for Evaluation of the Web-Based Course

The Web-based course will be evaluated using multiple sources of data such as course website content, assessment scores (quiz results), incoming student survey (1152 respondents; see Multimedia Appendix 1) and outgoing student survey (244 respondents; see Multimedia Appendix 2), dropout student survey (170 respondents; see Multimedia Appendix 3), semistructured staff interview (tutor and course director), and staff curricula vitae. Table 2 summarizes sources of data used for evaluation and the information provided.

**Table 1:** Program structure of the “Public Health Principles in Disaster and Medical Humanitarian Response” Web-based course.

Lesson number and topic		Assessment	Program milestones
1	Public Health Approaches to Medical Disaster Response	Quiz 1	Progress to lesson 3 after achieving 80.0% score
2	Disaster Concepts and Trends		
3	The Impact of Disasters	Quiz 2	Progress to lesson 4 after achieving 80.0% score
4	The Human Health Impact of Disasters	Quiz 3	Progress to lesson 5 after achieving 80.0% score
5	Responding to Health Needs in Disasters (I)	Quiz 4	Progress to lesson 6 after achieving 80.0% score
6	Responding to Health Needs in Disasters (II)		
7	Public Health Emergency Preparedness		
Lessons 1-7		Final quiz	Course completion certificate after achieving 60.0% score

**Table 2:** Sources of data for evaluation.

Source of data		Existing data	Information provided		Missing information
			Strength	Weakness	
**Data on the Web-based course**					
	Course website	Structure and format of the course	Enables benchmarking with criteria to describe what is sufficiently included and what is lacking in the course	No qualitative or quantitative data analysis	No data on students, staff, or outcomes
	Course assessment scores	Formative and final quiz results	Enables comparison with other programs	Evaluation of knowledge gained during the course only	No data on staff and student perceptions
**Data on students**					
	Incoming student survey	Student demographics	Standardized set of questions; high response rate (100.00%, 1152/1152); quantitative data analysis; provides information on student background; enables comparison with other programs	Does not directly evaluate the course	No data on staff and student perceptions or outcomes
	Outgoing student survey	Student perceptions	Standardized set of questions; quantitative and qualitative data analysis; enables comparison with other programs	Low response rate (21.00%, 244/1152); self-reported data; only students who completed the course participated; therefore, results are prone to bias	No data on staff perceptions
	Dropout student survey	Student perceptions	Standardized set of questions; quantitative data analysis; enables comparison with other programs	Low response rate (19.0%, 170/908); self-reported data; prone to bias because of low response rate	
**Data on staff**					
	Staff interview	Staff perceptions	Qualitative data analysis	Small sample size; cannot compare with other programs	No data on outcomes
	Curricula vitae of staff	Staff qualifications	Provides information on staff background; enables comparison with other programs	No quantitative or qualitative data analysis	No data on outcomes and student and staff perceptions

## Methods

### Evaluation Framework

Evaluation was based on the Donabedian model [9] and Greenhalgh et al’s quality framework for evaluating Web-based courses [10]. Table 3 summarizes the overlapping components of the frameworks.

### Six Criteria of Quality Framework by Greenhalgh and Colleagues

Following are the six criteria of Greenhalgh et al’s quality framework:

1. Course materials: Course materials will support the overall program aims, provide clear learning objectives, and promote active learning.
2. Interactive learning environment: Formal online discussions on key topics (virtual seminars) will support the overall program goals through high-quality, focused, academic discourse, collaboration, and lateral support.
3. Tutor performance and development: Module tutors will be appropriately qualified, trained, and supported to deliver high-quality learner support in the online environment.
4. Assessment: Assessment will be valid, reliable, fair, appropriate, efficient, timely, formative, and summative.
5. Student communication and support: The program will be supported by accessible, accurate, and up-to-date documentation. Support and advice will be tailored to the needs of individual students. There will be an effective system of student representation.
6. Administrative and technical support: Administrative and technical systems will support the program goals through high-quality service delivery, multidisciplinary teamwork, effective communication, and robust technological infrastructure. Administrative and technical staff will have clear roles and responsibilities and will be adequately supported in their work.

The Donabedian model [9], was originally developed to evaluate health care service programs but has also been adapted to evaluate courses with a Web-based component, such as blended learning [11]. In addition, it addressed practical outcomes such as whether the course reached the intended audience and cost. Greenhalgh et al’s quality framework was developed to assess a Web-based MSc program [10].

### Study Period

Study period was defined as the course period for the first cohort of students, that is, June 16, 2014 to December 15, 2014 (6 months).

### Study Subjects

Students who registered for the first cohort (n=1152) were included in the study. Two tutors were interviewed for assessing tutor performance and development, as well as administrative and technical support. The course director was interviewed when assessing the course cost. Verbal consent was obtained during interviews; all responses were anonymized; and permission was sought to review relevant documents such as course materials and surveys. This study fell under the auspices of learning and evaluation in the university and therefore did not need ethical approval.

### Data Source and Analysis

Table 4 summarizes the data source and analysis corresponding to the framework dimensions.

**Table 3:** Matrix of overlapping components of the evaluation frameworks.

Donabedian model	Greenhalgh et al’s framework
Structure and process	Course materials Interactive learning environment Tutor performance and development Student communication and support Administrative and technical support
Outcome Whether the course reached the intended audience Cost	Assessment

**Table 4:** Framework dimensions, data source, and analysis.

Framework dimension	Data source and analysis
Course materials	Examine course materials from CCOUC^a^ website Quantitative analysis of outgoing student survey: all students completing the final quiz responded (n=244)
Interactive learning environment	Examine interaction in the student discussion forum Semistructured interview with the course tutor Summarize relevant comments in the outgoing student survey
Tutor performance and development	Examine curricula vitae of the tutor Semistructured interview with the course tutor
Student communication and support	Quantitative analysis of incoming and outgoing student survey Examine course website for evidence of clarity, accuracy, and completeness of information on program content Examine course website for individualized summary of progress Semistructured interview with the course tutor
Administrative and technical support	Examine job descriptions and curricula vitae of staff in administrative and technical roles Examine funds allocated to administrative and technical support Summarize relevant comments in the outgoing student survey Semistructured interview with administrative and technical staff
Assessment	Examine assessment materials and methods on course website Quantitative analysis of assessment scores: assessment consisted of multiple-choice questions automatically graded by the compute Quantitative analysis of the outgoing student survey Semistructured interview with the course tutor
Whether the course reached the intended audience	Quantitative analysis of incoming and outgoing student survey Analysis of the dropout student survey
Cost	Interview with the course director

^a^CCOUC: Collaborating Centre for Oxford University and CUHK for Disaster and Medical Humanitarian Response.

## Results

### Course Materials

Overall program aims were as follows:

- Understand and discuss public health needs and gaps in disaster preparedness and response, specifically in the context of the Asia Pacific region.
- Systematically formulate key guiding questions during pre- and postdisaster phases to drive evidence-based disaster mitigation actions.
- Select and consult relevant and credible databases, guidelines, and documents to address the above issues.

Course materials supported the overall program aims by providing accessible online reading and multimedia materials. Reading materials were classified by level of difficulty, with optional “A-Closer-Look” text boxes to give additional context. A glossary was provided and a “Take-home Message” at the end of each section. Occasionally, students were directed to watch relevant videos on external websites. Clear learning objectives were provided at the start of the course and each chapter. Active learning was promoted by “Stop-and-Think” activities that posed a question, with answers behind a reveal button. There were polls for students to vote on a question and compare opinions.

**Figure 1:**
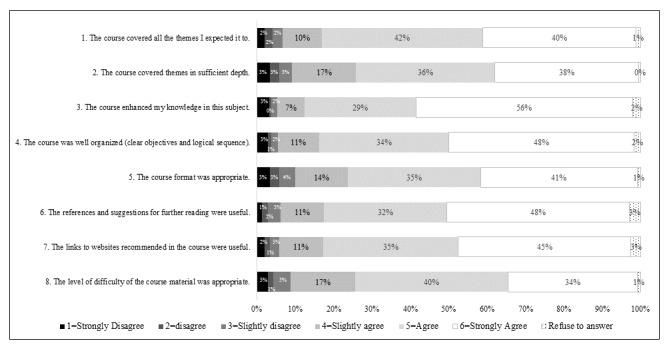
Student responses to statements regarding course content.

Figure 1 shows responses to the outgoing student survey, recorded on a Likert scale, from 1=strongly disagree to 6=strongly agree.

Most students answered positively regarding course content. In all 8 areas, over 90.0% (220/244) of students selected 4 or above (slightly agree, agree, or strongly agree). In the following 5 areas, over 80.0% (195/244) of students selected 5 or 6 (agree or strongly agree):

- The course covered all the themes I expected it to
- The course enhanced my knowledge (concepts and principles) in this subject
- The course was well organized (clear objectives and logical sequence)
- The references and suggestions for further reading were useful
- The links to websites or other parties/organizations recommended in the course were useful

**Figure 2:**
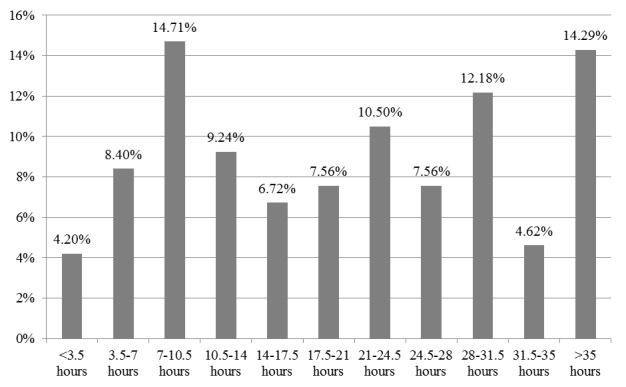
Number of hours students spent studying.

The course overview gave a clear estimation of workload, which was 1-3 hours per lesson, totaling 7-21 hours for 7 lessons. Figure 2 shows the total actual workload. Actual workload varied highly, from <3.5 hours to >35 hours. The most frequent responses were “7 hours to less than 10.5 hours” (14.7%, 36/244) and “35 hours or more” (14.3%, 35/244).

### Interactive Learning Environment

Content analysis of the online forum was conducted. There were no formal online discussions. However, each lesson had an informal discussion forum. Participation was voluntary and asynchronous. Students were free to create new threads on any topics. This was facilitated by the course tutor who occasionally read through threads and responded to questions. However, most of the content was grounded, drawing on students’ own experiences and course materials. For example, 1 student created a discussion thread on Ebola outbreak, which occurred during the course but was not covered in course materials. Others used the forum to reinforce learning of course materials by posting lesson summaries. Moreover, 6.60% of the students (76/1152) posted on the forum, generating 75 new threads and 216 posts.

Semistructured interview with the course tutor (who also served as the technical support officer) and subsequent content analysis revealed that there were no student complaints of difficulty in accessing the forum or problems with online etiquette. In the outgoing student survey, 1 question invited students to write any comments about the course or any specific suggestion as to how it could be improved: Several requested more online videos (eg, lectures) to supplement course materials, whereas only 1 student suggested the course could be improved by holding an online video conference.

### Tutor Performance and Development

Semistructured interviews were conducted with the program director and tutors. Questions were adapted from Greenhalgh et al’s quality framework criteria, standards, evidence, and quality failures. Subsequent content analysis was done according to these themes. The program director chose 2 tutors who also functioned as technical support officers throughout the course. Both were originally research assistants with Master of Public Health degrees who helped develop the course content. As there was no compulsory interaction on the course, tutors were only responsible for answering student queries and occasionally facilitating online discussion. This was done in addition to other roles and responsibilities that tutors had as research assistants for other projects. The program director appraised the tutors’ performance annually, although not specifically for the Web-based course. Tutor development was encouraged. One tutor is a PhD student and has been promoted to assistant lecturer, whereas the other has published in the *Lancet*.

Interview with the course tutor revealed that their workload was manageable and questions posed by students were within their capability. Students usually emailed the first tutor listed on the website. There were a few queries, ranging from course logistics to technical support. Students preferred to discuss academic questions among themselves in the forum, rather than asking the tutors. In the outgoing student survey, there were no complaints about the tutors or their level of input.

### Student Communication and Support

The course website clearly stated learning objectives for each lesson, expected time commitment, and required assessments to progress and gain a certificate. A personal progress log was available to each student. The course tutor’s contact details were listed for student queries. There were Web-based links to explore beyond course materials and an online forum for informal discussion. Student views were actively sought through a Web-based evaluation survey. In the outgoing student survey, 63.9% (156/244) had taken a similar course before, with 91.8% (224/244) rating the course as similar or better, and 93.9% (229/244) were satisfied with the course overall. Interview with the course tutor revealed that few students had queries regarding course navigation, material, or technical difficulties.

### Administrative and Technical Support

The program director selected course tutors to provide administrative and technical support, in addition to academic support. There was no dedicated training budget for tutors. In the outgoing student survey, there were no complaints regarding lack of administrative or technical support. Of 244 students, 181 (74.2%) preferred online learning over face-to-face learning. However, several students requested a PDF version to aid revision, as they lived in areas with suboptimal Internet access. Interview with course tutors revealed that there were few queries requesting administrative or technical support. All were within their capabilities.

### Assessment

Assessment consisted of Web-based multiple-choice questions completed anytime within the course. There were 4 short self-assessment quizzes and 1 final quiz for course completion. There were 10 questions in each short quiz. An 80.0% score was needed for students to proceed to the next lesson. Students were free to retake quizzes. As quizzes were drawn from a question bank, retake questions were not necessarily the same. The final quiz tested all course materials. A 60.0% score was required to achieve a certificate of completion. This method of assessment is reliable, as all questions are drawn from the same question bank and marked electronically. However, as there was no live monitoring during the quiz, it may not be a fair assessment, as it would be difficult to guard against cheating (eg, if someone else took the quiz in place of the student). This method has other advantages of being efficient, as minimal tutor time is required because of automatic computer marking. In addition, multiple formative quizzes allow students to have timely feedback on their progress.

The process of reviewing assessment questions was described in an interview with the course tutor. Course authors developed the assessment questions. These were reviewed by the program director, who is an international expert. Although course content was peer-reviewed by numerous academic colleagues, assessment questions were not reviewed by them, thereby decreasing content validity.

**Figure 3:**
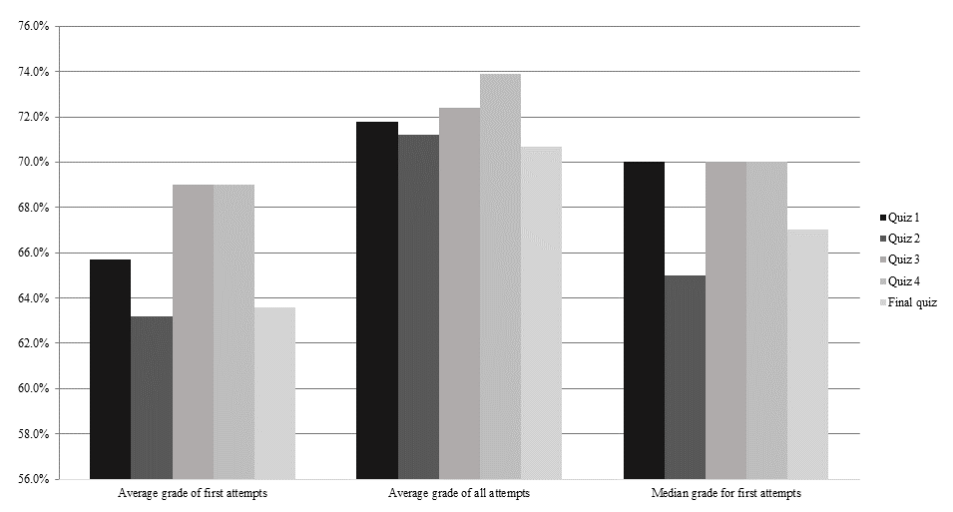
Average and median quiz grades.

Figure 3 shows average and median grades of all quizzes. Average grades for first attempts of the first 4 formative quizzes were between 63.2% and 69.0%. The average student needed to reattempt each quiz at least once to achieve the required grade to progress to the next lesson. The average grade for the first attempt of the final quiz was 63.6%, which would have been high enough for the average student to obtain the completion certificate (issued for grades of 60.0% or above) on their first attempt. Out of 1152 students registered in the cohort, 244 took the final quiz and 233 passed and gained a certificate of completion.

**Figure 4:**
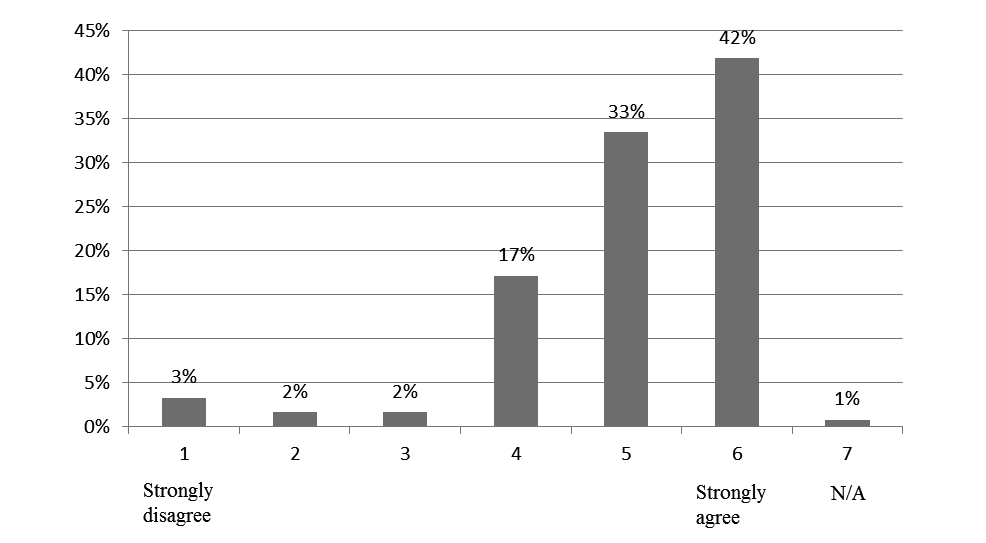
Students' responses to statement that assessment methods (quizzes) were appropriate. N/A: not applicable.

Figure 4 shows opinions on the assessment methods in the outgoing student survey. Most agreed that assessment methods of quizzes were appropriate. The scores and survey responses indicate an appropriate assessment, where course materials content is reasonably assessed in quizzes.

### Whether the Course Reached the Intended Audience

**Figure 5:**
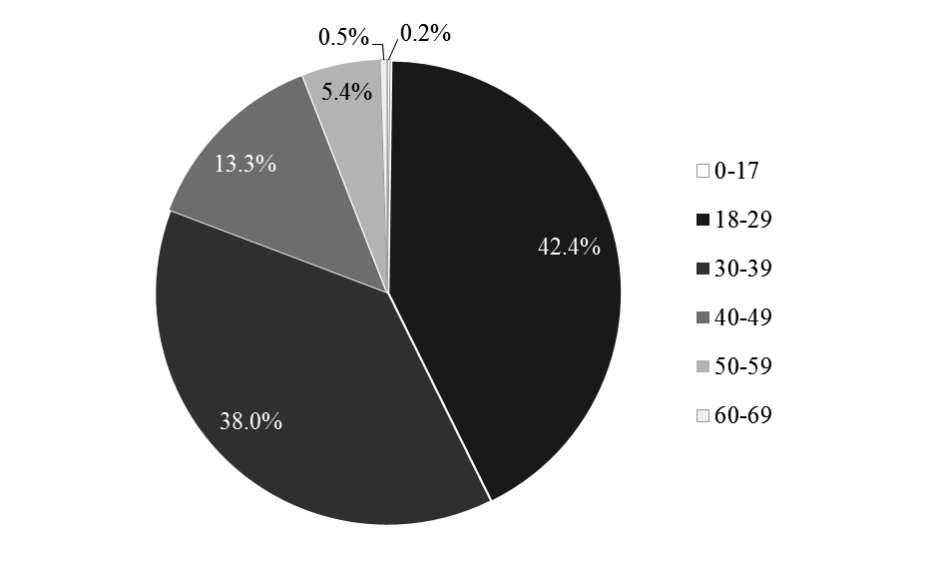
Student age (years).

**Figure 6:**
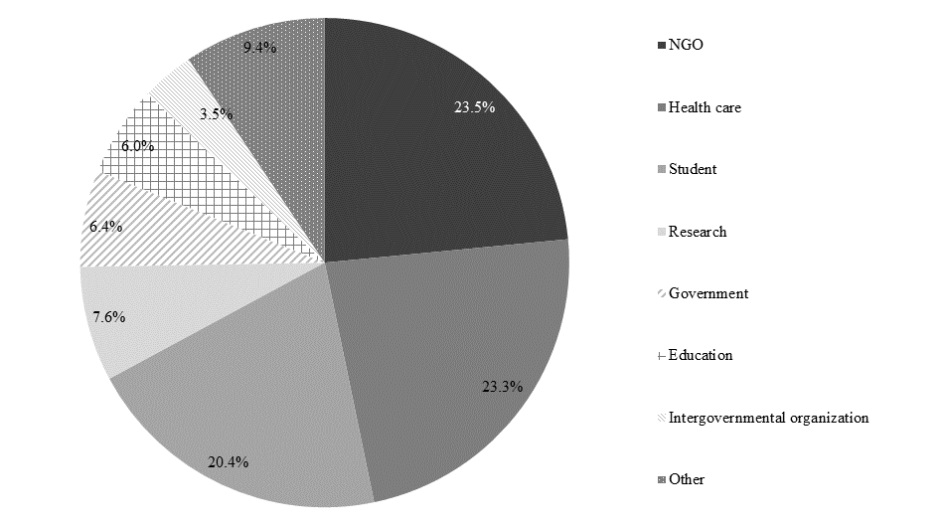
Student occupation.

**Figure 7:**
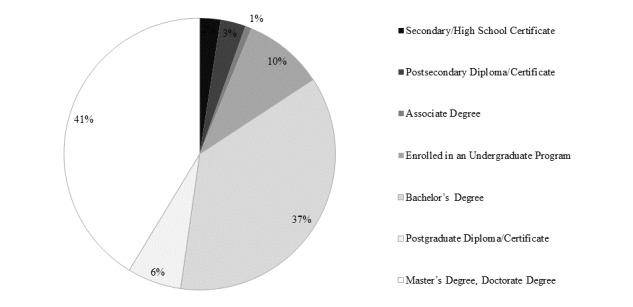
Students' highest academic qualifications.

**Figure 8:**
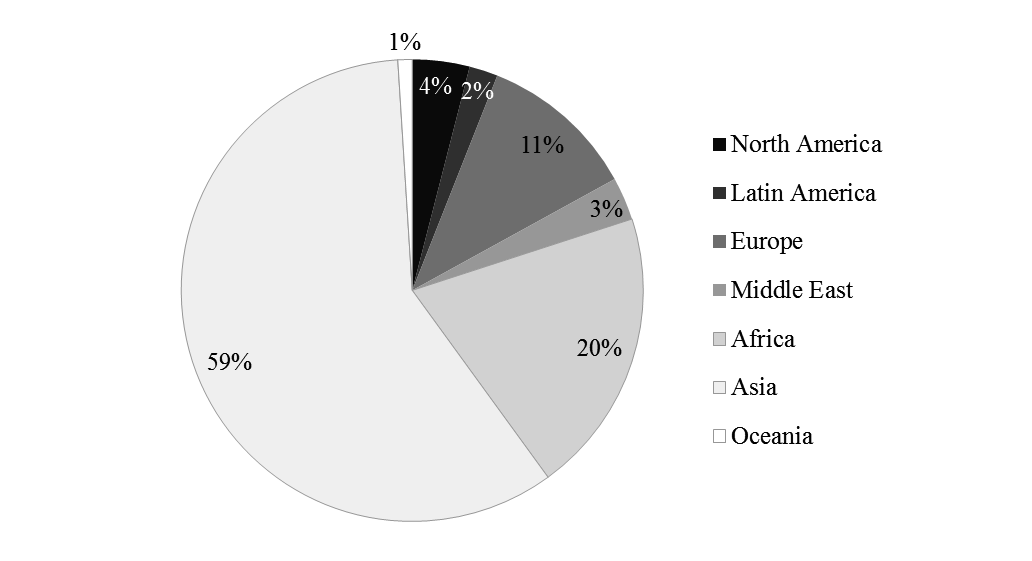
Students by continent.

The course was intended for postgraduates who are unable to access face-to-face learning. In a survey of all students (n=1152), the gender balance was roughly equal: 49.30% (568/1152) female, 50.50% (582/1152) male, and 0.00% (2/1152) who answered “others”). Figure 5 depicts student age. Most students were aged between 18 and 39 years. Figure 6 depicts students’ occupations. Most worked in nongovernmental organizations, health care sector, or were students. Figure 7 depicts highest academic qualifications obtained by students. Students’ highest academic qualifications were mostly a bachelor’s or a master’s degree. Figure 8 depicts students by continent. The majority of students came from Asia, with 41.00% being (472/1152) from Hong Kong.

The chi-square test comparing students who completed the course with dropouts showed no significant difference in gender (*P*=.40), age (*P*=.98), occupation (*P*=.43), or qualification (*P*=.17). Among those who completed the course, 48.7% (119/244) were local. A survey of 170 dropout students revealed that the main reasons for dropping out were change in schedule 71.2% (121/170) and lack of Internet access 25.9% (44/170). Moreover, 85.3% (145/170) would recommend the course to other people. As most students had at least a bachelor’s degree and were nonlocal (and therefore would have difficulties attending face-to-face learning), the course managed to reach the intended audience.

### Cost

Interview with the course director revealed that a grant of HK $109,000 was given to enroll 4000 students across 6 cohorts. This resulted in an average cost of HK $35 per enrolled student. In contrast, a face-to-face course with the same content at CUHK (The Chinese University of Hong Kong) charged a HK $513 enrollment fee.

## Discussion

### Principal Findings

Our study used Greenhalgh et al’s quality framework and the Donabedian model to assess the effectiveness of a Web-based course. This was done through content analysis of the course website, quantitative analysis of assessment scores and students’ surveys, semistructured interview with staff, and examining related administrative documents. Overall, the Web-based course was effective in using technology to deliver suitable course materials and assessment and to enhance student communication, support, and learning. It reached its intended audience of postgraduates who would have difficulties attending face-to-face learning, and the cost per student was much less compared with an equivalent face-to-face course.

The course materials supported the program aims by providing high-quality accessible reading and multimedia materials. These enabled students to “understand public health needs and gaps in disaster preparedness and response,” but not necessarily to “discuss” them; as there were no interactive tutorials or formal discussions, the interactive learning environment was dependent on informal interaction on the discussion board. Although this was used throughout the course, only 6.60% of students posted comments. In addition, to “formulate key guiding questions” and “select and consult relevant and credible databases, guidelines and documents to address the above issues” were part of the program aims. However, it would be difficult to develop these skills in depth during the course, as additional assignments (eg, exercises for group interaction or essay writing) would be needed to achieve these aims. Learning objectives were clearly stated and course materials promoted active learning, although not to the extent of fulfilling all program aims. Students were generally satisfied with the content and format of the course materials, suggesting that their personal aims might be less ambitious than the program aims. Additional assignments would require more tutor resources. Peer grading has been advocated in MOOCs, but suffers from difficulty in quality control [12]. In addition, the funnel of participation might further narrow, resulting in less students participating and completing the course [13]. Modifying the program aims to align with student aims might be more realistic than increasing active learning to achieve the current program aims.

One of the quality failures listed in the quality framework [10] noted that “poor performance by a majority of students on a course should raise questions about course design or tutor competence, whereas poor performance by a minority of students is usually attributable to other factors.” As average performance in assessments was reasonably satisfactory, it could be concluded that course design and tutor competence were adequate. Course tutors were familiar with the course materials, as their research was in similar areas. However, tutors played a passive role in the course: students sought out tutors infrequently, and tutors monitored and occasionally participated in organic discussions. This approach to teaching has also been used in other MOOCs [14]. In a face-to-face setting, tutors could identify struggling students by inattention or lack of attendance, with early intervention to improve learning. However, identifying these students in a Web-based environment is difficult, especially with large enrollment numbers. This may account for the low completion rate of MOOCs [7]. Current research aims at using student engagement on the Web to identify those who are struggling [15], which may improve completion rates while posing less additional burden on the tutor.

Assessment questions would have improved content validity if peer-reviewed by experts. Assessment using Web-based quiz was reliable, although not necessarily as fair as face-to-face assessment. The Web-based quizzes were appropriate, efficient, and timely and included both formative and summative assessment. The assessment format may have been conducive to the relatively high course completion rate (20.00%, 230/1152) as compared with MOOCs with completion rates of 7.0% to 9.0% [6]: the quizzes had flexible quiz deadlines and did not combine other assessment methods. One study comparing multiple MOOCs showed that courses with flexible deadlines had higher course completion rates (15.5%) compared with those with firm deadlines (4.6%). In addition, courses with solely Web-based quizzes as assessment methods had a higher course completion rate (14.9%) compared with those combining quizzes with other assessment methods such as peer assessment (7.9%) [14]. Another study reported that the result of multiple Web-based quizzes throughout the course was the strongest explanatory variable in final exam scores when compared with other assessment methods (eg, self- and peer assessment) [16].

Participants had similar backgrounds to those in other MOOCs; they were mostly young, well educated, and employed. However, they differed in gender and location. One study reported 56.9% of male participants across 32 MOOCs, whereas the Web-based course had 49.30%. In addition, most students at the University of Pennsylvania’s MOOCs came from the United States or non-US OECD (Organisation for Economic Co-operation and Development) countries [17]. In contrast, students in the Web-based course mostly came from Asia, with 41.00% (472/1152) from Hong Kong. This reflects the tendency of Web-based courses to attract participants locally as well as internationally. As most students were international (59.00%, 680/1152), the Web-based course effectively reached a diverse population who did not have easy access to a face-to-face course in Hong Kong.

Participant demographics point to a cohort who would likely be more self-motivated and technology literate than the general population. Most students in the Web-based course had at least a bachelor’s degree, which coincided with the course’s target audience, who were postgraduate-level public health practitioners working in the field of disaster management. Adequate learning infrastructure might provide sufficient support to these students. The Web-based course provided this infrastructure through resources on the Web, forums, and a “check your progress” tool. Most outgoing students reported satisfaction with the course, and there were no complaints regarding the level of communication and support provided. In addition, students dropped out mainly because of personal reasons. A higher level of student support could be provided by using the “check your progress tool” to alert the staff about students who are progressing poorly through the course.

Despite the lack of specific staff training budget, students who completed the course were generally satisfied with the administrative and technical support. This may be related to the choice of online platform. Moodle was used, with the advantage of being free and widely used globally [18]. In addition, the bulk of course material was in written format, enabling easier access to those with lack of high-speed Internet. Although some students suggested including video lectures, using this format would have increased access difficulty, which increased technical support may be unable to solve. On the other hand, some students requested a PDF version so as to increase access to the course material when the Internet was not available. Studies analyzing video use in MOOCs reported that only half of participants and certificate earners watched the majority of course videos [19,20]. Despite participants from developing countries such as India earning the most certificates compared with other countries, use of video lectures was conversely lower. One explanation was that poor Internet access was an obstacle to downloading videos [21]. Another study conducted in fragile contexts, which the Web-based course aims to reach, noted that video lectures were an insurmountable obstacle and that downloaded materials were widely shared locally, enabling a wider reach of learners than those who initially accessed the material on the Web [22]. The conflicting student comments from the Web-based course reflect a range in preferred learning styles and ease of technological access. Including video lectures would require more financial resources, yet appeal to more people. However, changing the format from written course material to video lectures might result in excluding those with limited Internet access. The lack of request for online video conference or live tutorial discussions could be because of the required technology, extra time commitment, and difference in student time zones, which may discourage participants.

The cost of the course was reasonable, with the Web-based course costing significantly less than a comparable face-to-face course.

### Implication of Findings

Using the Donabedian model [9] and Greenhalgh et al’s [10] structured quality framework enabled this evaluation to identify strengths and weaknesses that would be omitted in a conventional evaluation that assessed outcomes only. The resulting comprehensive approach to evaluation will be useful for providing recommendations to improve the program.

### Limitations

Ideally, the Web-based course should have multiple independent reviewers to evaluate the program. However, in this report, there is only 1 reviewer (the author). As it was difficult to find frameworks to adequately evaluate free Web-based courses, the quality framework used was originally developed from evaluation of a Web-based MSc course. These Web-based MSc courses are accredited, fee paying, and last up to 5 years [23]. In contrast, the course is nonaccredited, free, and lasts for 6 months. Thus, some of the criteria were difficult to apply to the course, as the course would have less resources and educational time when compared with the Web-based MSc course.

As the Web-based course attracted students globally, it was not possible to evaluate the effectiveness of the program using a randomized controlled design, which would have compared the Web-based course to face-to-face learning. In addition, students came from a range of backgrounds, and each individual’s learning experience varied. Although all incoming students answered a questionnaire concerning demographics, other aspects of evaluation dealt with students’ experience on the course. Therefore, only those who completed the course answered the outgoing questionnaire. This may have resulted in possible overestimation of course satisfaction, as those who were dissatisfied may have dropped out early. In addition, the dropout student survey had a low response rate of 19.0% (170/908).

### Recommendations

The program aims could be modified to align with student expectations and to reflect what could be achieved realistically with limited time and resources in a free, 6-month Web-based course. Moreover, to increase accessibility to course materials, a PDF version of the course material could be made available for download. Accompanying material such as books, CD-ROM (Compact Disc Read-Only Memory), and video lectures of the course material could supplement the existing course. These could potentially be available at a price, to subsidize the staff and technological infrastructure needed to run the course. Additional options for students who completed the course would enable them to continue their education. Students who completed the course could be invited to attend a fee-paying, face-to-face, short course to facilitate active learning, such as discussions and essay assignments. Completion of the face-to-face course could lead to university credit. Using the Web-based course as part of requirements to gain credit would motivate students who desire advanced learning and accreditation. Struggling students could be identified by tracking their progress and engagement on the Web. These students could be automatically emailed to ask what problems they may be encountering. This would proactively identify which students may need a tutor’s help. Finally, assessment questions would have higher content validity if peer-reviewed by experts.

### Barriers to Implementation of Recommendations

Producing supplementary material would require additional resources. However, students may be unwilling to pay for the supplementary material or additional face-to-face courses. Necessary software would also be needed to track student progress and send automatic alerts. However, this may not be widely available yet and may pose additional costs to the course.

### Conclusions

The “Public Health Principles in Disaster and Medical Humanitarian Response” Web-based course is effective in using technology to deliver suitable course materials and assessment and to enhance student communication (via discussion boards), support (via access to staff), and learning (using interactive Web-based tools). It is also effective in reaching the intended audience. However, there are a few areas for improvement. Program aims could be modified to align with student aims, while supplementing with increased active learning (eg, video lectures, essay writing, live tutorials, and discussions) for those who desire further learning. Assessment questions could be reviewed by experts. In addition, active efforts could be made to identify struggling students and to provide better support.

## Appendix

Multimedia Appendix 1 Incoming student survey.

Multimedia Appendix 2 Outgoing student survey.

Multimedia Appendix 3 Dropout student survey.

## Abbreviations

CD-ROM: Compact Disc Read-Only Memory
MOOC: massive open online courses
OECD: Organisation for Economic Co-operation and Development
