# Supplementary material for: A Web-Based Course on Public Health Principles in Disaster and Medical Humanitarian Response: Survey Among Students and Faculty
Source: JMIR Med Educ. 2018 Jan 26;4(1):e2. doi: 10.2196/mededu.8495 (PMC5807623; doi:10.2196/mededu.8495)
Supplement: Multimedia Appendix 1 [file mededu_v4i1e2_app1.pdf]

Background information

Incoming student survey

Age*

Gender*

Required

Choose...

Citizenship (If you have dual citizenship please select the country you reside/spend most time in)*

Required

Choose...

Country of residence*

Required

Choose...

First language*

Required

Choose...

Highest academic qualification (This online course is intended for students and holders of a Bachelor's Degree, as well as secondary/high school graduates with at least three years of work experience.)*

Required

Choose...

Name of school/university where the highest academic qualification was obtained*

Field of the highest academic qualification obtained*

Required

Choose...

Current occupation*

Choose...
