# Supplementary material for: A Web-Based Course on Public Health Principles in Disaster and Medical Humanitarian Response: Survey Among Students and Faculty
Source: JMIR Med Educ. 2018 Jan 26;4(1):e2. doi: 10.2196/mededu.8495 (PMC5807623; doi:10.2196/mededu.8495)
Supplement: Multimedia Appendix 2 [file mededu_v4i1e2_app2.pdf]

Outgoing student survey

PART 1 – Course Content

	Strongly Disagree					Strongly Agree	N/A
1. The course covered all the themes I expected it to.	1	2	3	4	5		**N**
2. The course covered themes in sufficient depth.	1	2	3	4	5		**N**
3. The course enhanced my knowledge (concepts/principles) in this subject.	1	2	3	4	5		**N**
4. The course was well-organized (clear objectives and logical sequence).	1	2	3	4	5		**N**
5. The course format was appropriate (i.e. right mix of text, activities, quizzes, etc.).	1	2	3	4	5		**N**
6. The references and suggestions for further reading were useful.	1	2	3	4	5		**N**
7. The links to websites of other parties/organisations recommended in the course were useful.	1	2	3	4	5		**N**
8. The level of difficulty of the course material was appropriate.	1	2	3	4	5		**N**

PART 2 – Quizzes

	Strongly Disagree					Strongly Agree	N/A
9. Assessment methods (quizzes) were appropriate.	1	2	3	4	5	6	**N**

Part 3 – Overall evaluation

10. When compared with similar courses in delivery format (i.e. online) and topic (i.e. disaster-related) you have taken, how would you rate this course?

Worst ever taken	Worse than average	Similar	Better than average	Best ever taken	Not applicable, I've never taken this kind of course before	I prefer not to answer
1	2	3	4	5	**N1**	**N2**

Part 4 – General

11. Approximately how many hours did you spend studying the course materials?

1	2	3	4	5	6	7	8	9	10	11
Less than 3.5 hours	3.5 hours to less than 7 hours	7 hours to less than 10.5 hours	10.5 hours to less than 14 hours	14 hours to less than 17.5 hours	17.5 hours to less than 21 hours	21 hours to less than 24.5 hours	24.5 hours to less than 28 hours	28 hours to less than 31.5 hours	31.5 hours to less than 35 hours	35 hours or more

12. Are you interested in taking a follow-on course on public health and disaster response? If so, what is the delivery format you prefer for this follow-on course?
    1) No, I am not interested.
    2) Yes, I am interested and I prefer a face-to-face course.
    3) Yes, I am interested and I prefer an online course.
