# Supplementary material for: A Web-Based Course on Public Health Principles in Disaster and Medical Humanitarian Response: Survey Among Students and Faculty
Source: JMIR Med Educ. 2018 Jan 26;4(1):e2. doi: 10.2196/mededu.8495 (PMC5807623; doi:10.2196/mededu.8495)
Supplement: Multimedia Appendix 3 [file mededu_v4i1e2_app3.pdf]

Drop-out students' survey

1. What were the reasons that made you discontinue studying this course? (You can choose more than one reason.)

- [ ] The content is too difficult. The content is
- [ ] too simple. The content is too long.
- [ ] The content is too short. The quizzes are
- [ ] too difficult.
- [ ] The organization and flow of the content are inappropriate. The design
- [ ] and layout of the course website is unattractive.
- [ ] The format of the course (a mix of texts and images without video) is inappropriate. I had difficulty
- [ ] accessing the internet.
- [ ] My schedule had changed, which prevented me from continuing the study. If you have other
- [ ] reasons or any other comments, please specify below:

Multiple Lines

---

2. Will you recommend this course to your colleagues, students or friends?

- ( ) Yes
- ( ) No
- ( ) Maybe
